# EIF2S2 is a novel independent prognostic biomarker and correlated with immune infiltrates in hepatocellular carcinoma

**DOI:** 10.3389/fgene.2022.992343

**Published:** 2022-10-05

**Authors:** Jing Liu, Tongyu Liu, Chuanhao Zhang, Jiabei He, Dong Zhou, Zhe Wang, Ruoyu Wang

**Affiliations:** ^1^ Department of Medical Oncology, Affiliated Zhongshan Hospital of Dalian University, Dalian, China; ^2^ The Key Laboratory of Biomarker High Throughput Screening and Target Translation of Breast and Gastrointestinal Tumor, Dalian University, Dalian, China; ^3^ Department of Gynecology, Fujian Cancer Hospital, Fujian Medical University Cancer Hospital, Fuzhou, China

**Keywords:** EIF2S2, HCC, prognosis, immune infiltration cells, drug sensitivity

## Abstract

**Background:** Hepatocellular carcinoma (HCC) is a highly malignant disease with poor prognosis. It is urgent to find effective biomarkers. Eukaryotic Translation Initiation Factor 2 Subunit Beta (EIF2S2) is a subunit of heterotrimeric G protein EIF2, and its function is still unclear. We studied the role of EIF2S2 in the malignant progression of liver cancer and its relationship with immune infiltration.

**Methods:** Download the RNA expression and clinical information of EIF2S2 from the Cancer Genome Atlas (TCGA) database, analyze the relationship between the expression of EIF2S2 and the prognosis and clinicopathological characteristics of HCC, analyze the differential genes by Gene Ontology (GO), Kyoto Encyclopedia of Genes and Genomes (KEGG) and tumor related immune infiltrating cells. The Protein expression level of EIF2S2 was obtained from Human Protein Atlas (HPA) databases. The relationship between EIF2S2 expression and immune infiltrates in HCC was analyzed on TIMER 2.0. The data processing analysis based on R language. Drug Sensitivity data from Genomics of Drug Sensitivity in Cancer (GDSC).

**Results:** EIF2S2 is highly expressed in HCC patients and is associated with poor prognosis. The expression of EIF2S2 was also correlated with age, clinical stage and pathological grade. Univariate and multivariate COX regression analysis showed that EIF2S2 was an independent risk factor for survival. The receiver operating characteristic (ROC) curve of EIF2S2 also confirmed the diagnostic value of EIF2S2 in HCC patients. Through GO and KEGG enrichment analysis, EIF2S2 expression was found to be closely related to some immune pathways. The expression of EIF2S2 was correlated with memory B cell, plasma B cell, CD8+ T cell, CD4+ resting memory T cell and the expression of some immune checkpoints, such as PDCD1, TIGIT and CTLA-4. It is also more sensitive to paclitaxel, sunitinib and other drugs.

**Conclusion:** This study shows that EIF2S2 can be used as a prognostic factor for HCC, which is closely related to immune infiltration and immune checkpoints, and may play a potential regulatory role in predicting drug sensitivity.

## Introduction

HCC causes more than 700,000 deaths worldwide every year, and has become the sixth most common tumor in the world. The incidence of HCC varies from region to region, with the highest incidence in Asia, followed by Europe and Africa. And HCC is also the fourth leading cause of cancer death after lung, colorectal and gastric cancer (Singal et al., 2020). Clinically, the overall incidence of HCC is heterogeneous, which may be due to differences in hepatitis virus prevalence and environmental factors. Around the world, the incidence of HCC in men is two to three times that in women, and the difference is even greater in high-incidence areas (Samant et al., 2021). Chronic hepatitis B virus infection is a major risk factor for the development of cirrhosis and HCC, while other important risk factors include hepatitis C virus infection, alcoholic liver disease, and non-alcoholic fatty liver disease (Ganne-Carrie and Nahon, 2019; Villanueva, 2019; Younossi and Henry, 2021).

Despite various treatment options for HCC, including surgery, interventional therapy, immunotherapy and targeted therapy, the 5-year overall survival rate of patients with HCC remains unsatisfactory, mainly because of the high tumor recurrence and metastasis rates (Kanno et al., 2021; Llovet et al., 2021). Due to the lack of early detection strategies and effective treatment, the 5-year survival rate of HCC is still as low as < 12% (Hlady et al., 2019).

Therefore, it is of great importance to discover new molecules related to the progression of HCC, identify new diagnostic markers and therapeutic targets, accurately select immune checkpoint inhibitors in the population, find the beneficiaries of immunotherapy for improving the prognosis of HCC patients.

EIF2S2 is a subunit of EIF2, a heterotrimeric G protein composed of *α*, *β* and *γ* subunits. Eukaryotic cells restrict protein synthesis under various stress conditions by inhibiting EIF2S2 (Kashiwagi et al., 2016). Previous studies have shown that EIF2S2 deletion reduces the incidence of human testicular germ cells in a mouse model of testicular germ cell tumor development, which also suggests that EIF2S2 is involved in cell proliferation and differentiation (Heaney et al., 2009). Studies have shown that the PI3K/Akt/GSK-3β/ROS/EIF2S2 pathway could regulate NK cell activity and tumor cell sensitivity to NK cells, leading to breast cancer growth and lung metastasis (Jin et al., 2019). It has also been shown that EIF2S2 is highly expressed in gastrointestinal cancers and can promote cell proliferation, migration and colorectal cancer invasion (Zhang et al., 2020). At the same time, EIF2S2 gene is also found to be highly expressed in lung cancer, which can predict the prognosis of lung adenocarcinoma patients (Tanaka et al., 2018). All of the above indicate that EIF2S2 plays an important role in the occurrence, development and metastasis of cancers. However, there is no report on the correlation between EIF2S2 and HCC prognosis and immune infiltration.

In this study, we found that EIF2S2 was highly expressed in HCC, and high expression was associated with poor prognosis and clinicopathological features. We also found that the expression of EIF2S2 was related to a variety of immune infiltrating cells such as T cell CD8+, T cell CD4+ memory resting and a variety of immune checkpoint, such as PDCD1, TIGIT, CTLA4. And it is highly sensitive to paclitaxel, sunitinib and other drugs. Our study shows that EIF2S2 can be used as a prognostic marker in HCC and is associated with immune infiltration. This is the first study of the expression of EIF2S2 in HCC, which may help clinically discover and understand the related processes and potential treatments of EIF2S2 expression in HCC target.

## Materials and methods

### Data acquisition and processing

The liver hepatocellular carcinoma mRNA expression data and corresponding clinical information were obtained from the TCGA database (https://portal.gdc.cancer.gov/), which was used to evaluate the expression of EIF2S2 in 374 liver hepatocellular carcinoma samples and 50 adjacent normal samples. HCC patients were divided into high expression group and low expression group according to the median value of EIF2S2 expression. Data were collected and analyzed using R 4.1.3 software. And the expression data of EIF2S2 in different types of tumors were obtained from the TIMER2.0 database (http://timer.comp-genomics.org/) (Li et al., 2020). The Human Protein Atlas (HPA) database (https://www.proteinatlas.org/) is a public database that downloads the protein expression levels of EIF2S2 in normal and HCC tissues from HPA.

### Survival analysis and clinicopathological analysis

Survival data were statistically analyzed by the survivor R software package, and 374 HCC samples were visualized by the “survminer” R software package. EIF2S2 mRNA expression and its correlation with overall survival (OS) and progression-free survival (PFS) in patients with HCC were analyzed using the TCGA–LIHC dataset. The time-dependent receiver operating characteristic (ROC) curve was drawn using the “timeROC” package (Robin et al., 2011) of R software to evaluate the specificity and sensitivity of EIF2S2 expression for the prognosis assessment of HCC. In addition, the correlation between EIF2S2 expression and clinicopathological factors was also analyzed.

### Independent prognostic analysis

Univariate and Multivariate Cox risk regression analysis of EIF2S2 were performed to confirm whether EIF2S2 and clinicopathological parameters were independent factors associated with HCC.

### Eukaryotic translation initiation factor 2 subunit beta co-expression analysis

The RNA data obtained from the TCGA database were used to screen out differentially expressed genes between the high EIF2S2 subgroup and the low EIF2S2 subgroup. Analyze the genes that have a co-expression relationship with EIF2S2, understand the positive and negative regulatory relationship between EIF2S2 and genes, and draw heat map according to differential genes.

### Enrichment analysis

Differentially expressed genes were subjected to GO and KEGG analysis, and the “ClusterProfilter” R software package was used for GO and KEGG enrichment analysis (Yu et al., 2012), and significant enrichment pathways were obtained. The GO analysis included cellular composition (CC), molecular function (MF) and biological process (BP).

### Immune cell infiltration and immune checkpoint analysis

We used TIMER2.0 to evaluate the correlations between the expression level of EIF2S2 and the infiltration levels of immune cells with all algorithms provided, like EPIC, TIMER, CIBERSORT, CIBERSORT-ABS, QUANTISEQ, XCELL, and MCPCOUNTER algorithms. In addition, the correlation between EIF2S2 expression and immune checkpoints was evaluated with Spearman’s correlation test.

### Drug sensitivity analysis

Based on the largest pharmacogenomics database Genomics of Drug Sensitivity in Cancer (GDSC), Home page - Cancerrxgene - Genomics of Drug Sensitivity in Cancer, we used the R package “pRRophetic” to predict the chemotherapy sensitivity of each tumor sample (Geeleher et al., 2014). Regression methods were used to estimate IC50 for each specific chemotherapeutic drug, predicting the relationship between EIF2S2 and sensitivity to different drugs.

### Statistical analysis

The statistical analysis R packages used in each step of the statistical analysis are mentioned above. All data analyses were carried out on R version 4.1.3. Kaplan-Meier curves were used to estimate OS in different groups, and differences between curves were analyzed by log-rank test. Hazard ratios (HR) and 95% confidence intervals (CI) were estimated using univariate and multivariate Cox regression models, and *p* values < 0.05 were considered statistically significant. *p < 0.05, **p < 0.01, ***p < 0.001.

## Results

### Eukaryotic translation initiation factor 2 subunit beta is highly expressed in hepatocellular carcinoma

**FIGURE 1:**
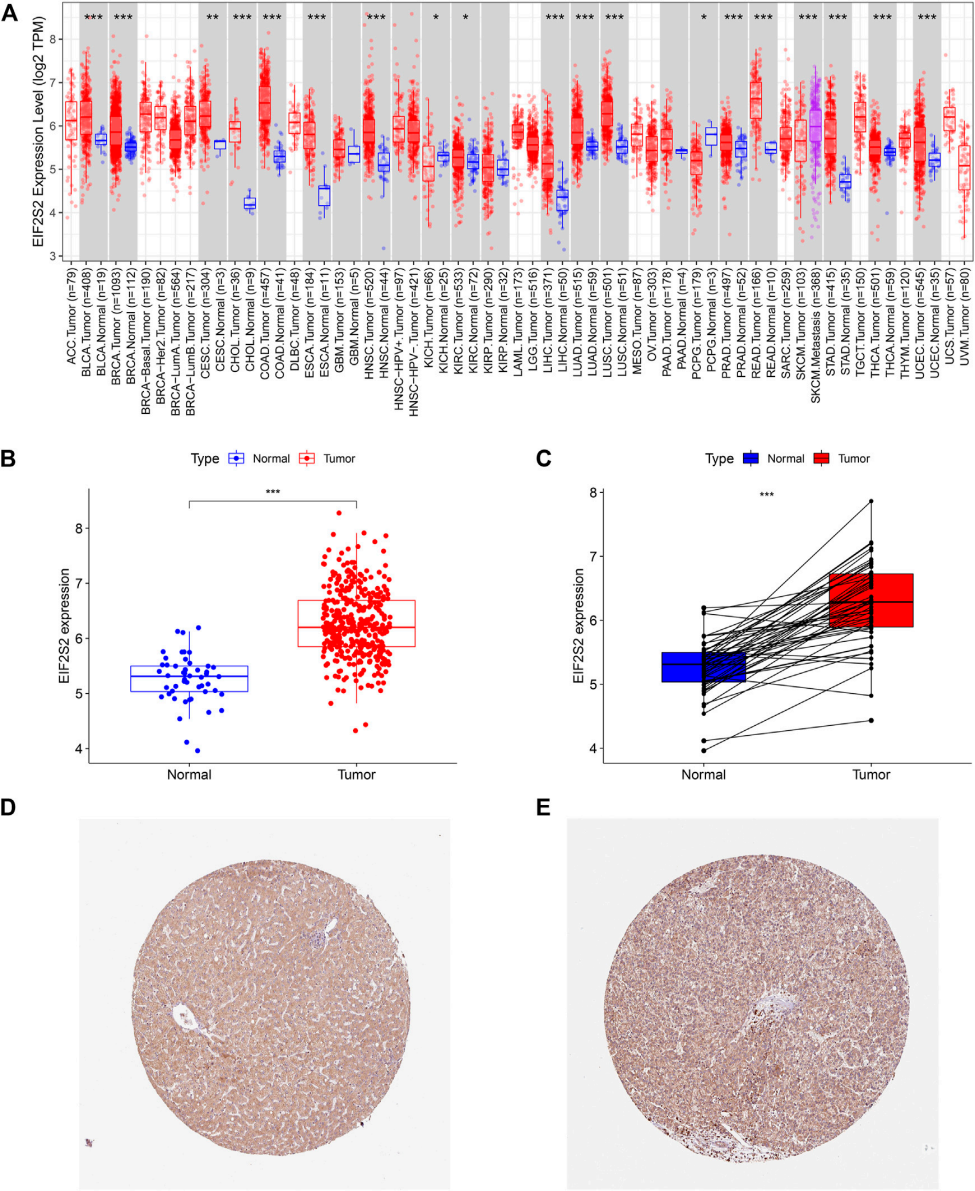
The expression of EIF2S2 in HCC. **(A)** EIF2S2 expression levels in different human tumor types according to TIMER 2.0 database. **(B)** EIF2S2 expression was compared in tumor tissue and control tissue samples in the TCGA database. **(C)** EIF2S2 expression levels were compared for paired tumor and control tissue from the TCGA database. **(D,E)** Representative immunohistochemical images of EIF2S2 protein expression in normal and hepatocellular carcinoma tissues. *p < 0.05, **p < 0.01, ***p < 0.001.

The TIMER 2.0 database showed that compared with normal samples, EIF2S2 was overexpressed in most types of cancer, including HCC, colon cancer, lung adenocarcinoma, prostate cancer, endometrial cancer, breast invasive cancer, etc. (Figure 1A). Focusing on HCC, we examined the expression of EIF2S2 in 374 HCC tissues and 50 adjacent normal tissue from the TCGA database. The results showed that the mRNA expression of EIF2S2 was significantly upregulated in HCC tissues compared with adjacent normal tissues (*p* < 0.001) (Figure 1B). Similarly, the expression of EIF2S2 mRNA in cancer samples was also significantly increased in 50 paired cases of HCC tissues and adjacent normal tissues (*p* < 0.001) (Figure 1C). Furthermore, we analyzed immunohistochemical samples from HPA, which confirmed the level of EIF2S2 expression was higher in HCC tissue than in normal samples (Figures 1D,E).

### Eukaryotic translation initiation factor 2 subunit beta expression is associated with poor prognosis in hepatocellular carcinoma

**FIGURE 2:**
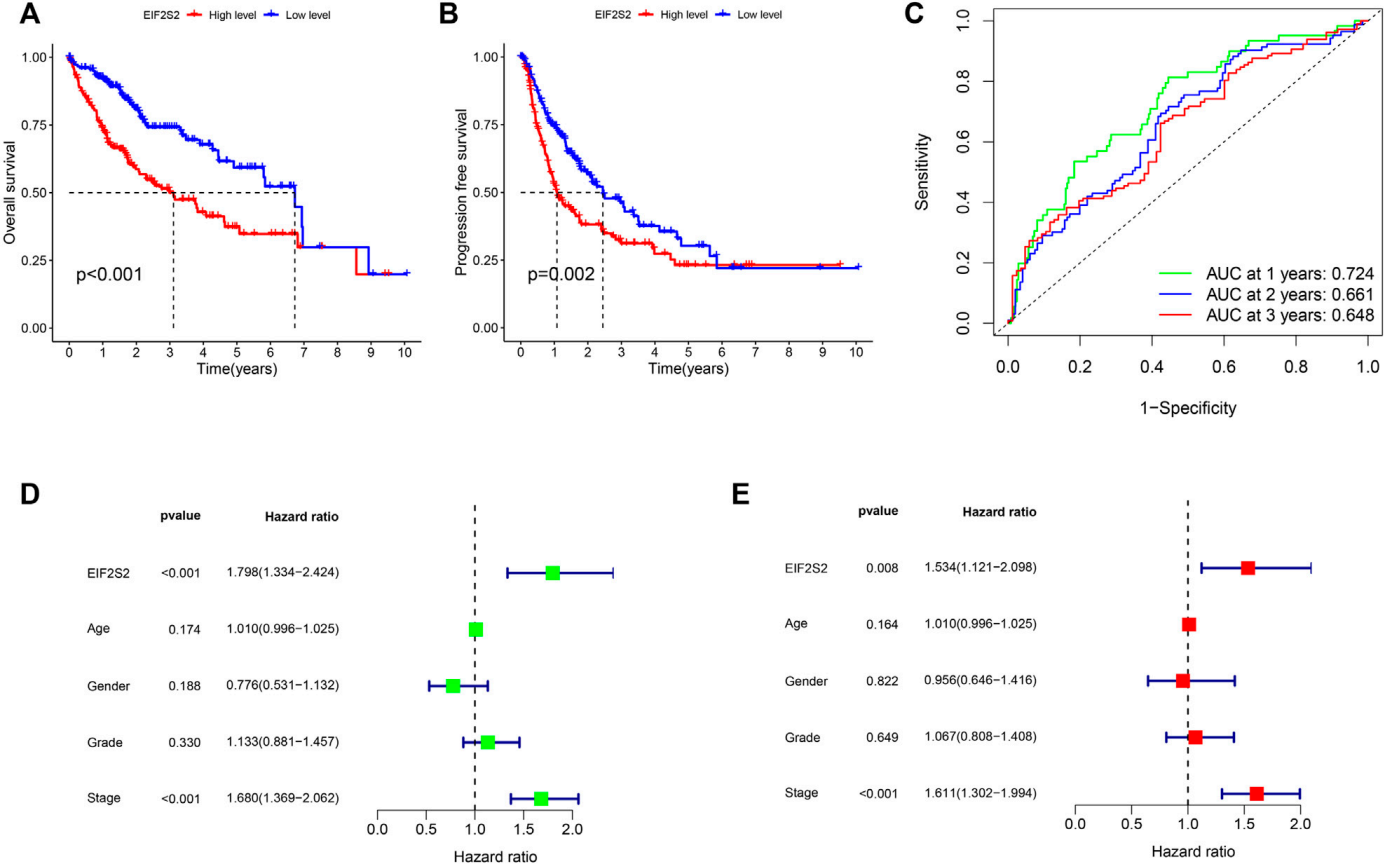
Survival and prognosis analysis of EIF2S2. **(A)** High EIF2S2 expression groups had a shorter OS. **(B)** High EIF2S2 expression groups had a shorter PFS. **(C)** AUC of time-dependent ROC curves to evaluate the predictive efficacy of the prognostic signature for OS in HCC patients **(D)** EIF2S2 could act as a risk factor for survival with Multivariate cox regression analysis. **(E)** EIF2S2 could act as a risk factor for survival with Univariate cox regression analysis.

To further investigate the prognostic value of EIF2S2 expression in HCC, we used Kaplan-Meier curve to analyze the relationship between EIF2S2 expression and OS and PFS of HCC patients. The results showed that the OS and PFS of HCC patients with high EIF2S2 expression were inferior to those of HCC patients with low EIF2S2 expression (Figures 2A,B). To determine the predictive value of the EIF2S2 expression signature, an ROC curve for 3-year survival was constructed based on the optimal critical risk value. EIF2S2 has good accuracy in predicting the 1-year OS of HCC, and larger samples may be needed to verify 2-year and 3-year OS in the future (Figure 2C). We performed univariate and multivariate independent prognostic analyses of EIF2S2 expression. The forest plot demonstrates that EIF2S2 expression was considered as risk factors affecting the prognosis of HCC patients in univariate and multivariate Cox regression (Figures 2D,E).

### Correlation of eukaryotic translation initiation factor 2 subunit beta expression with clinicopathological features

**FIGURE 3:**
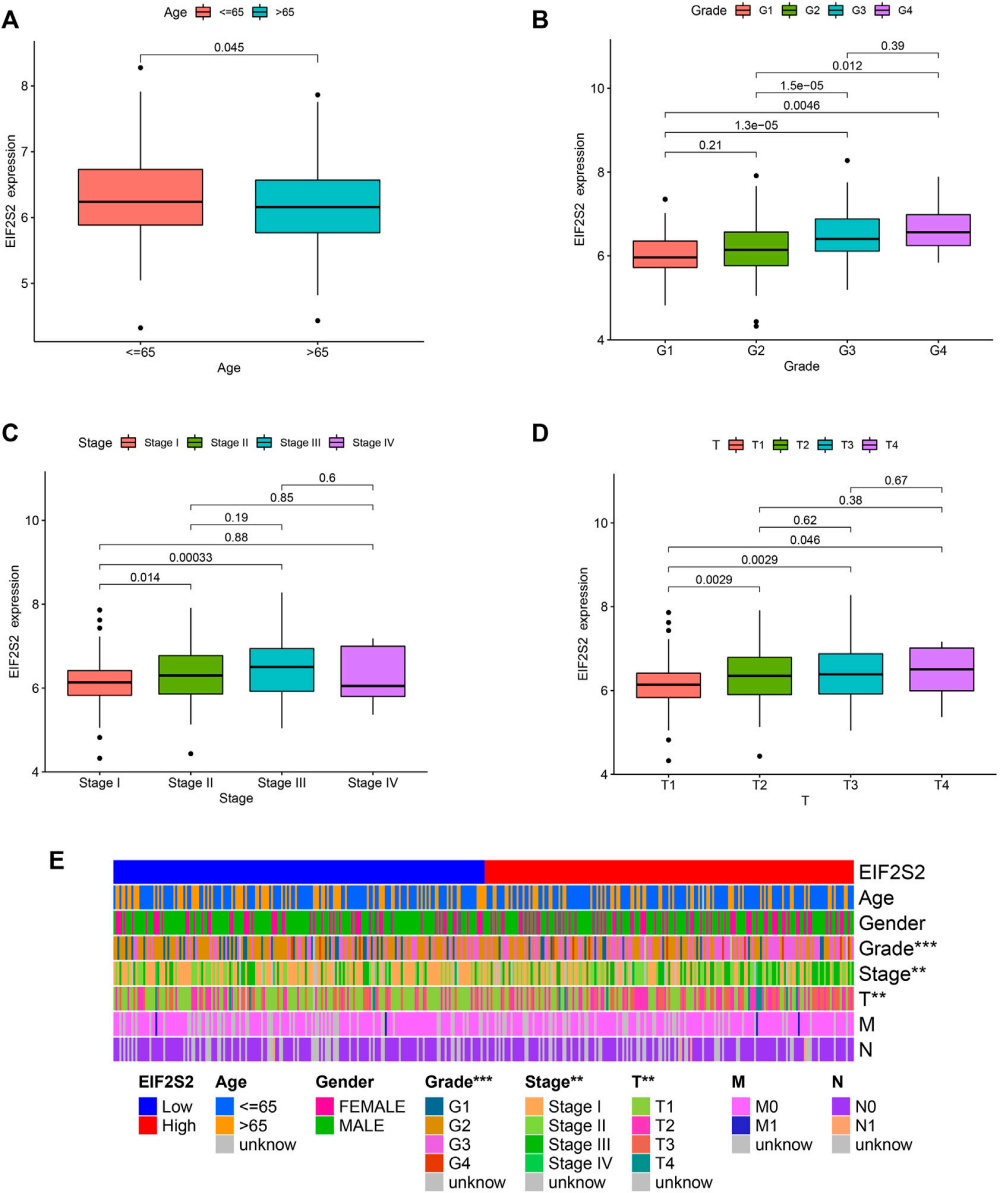
Association between the EIF2S2 expression and different clinicopathologic characteristics. **(A)** Association between the EIF2S2 expression and the age of HCC. **(B)** Association between the EIF2S2 expression and the histologic grade of HCC. **(C)** Association between the EIF2S2 expression and the pathologic stage of HCC. **(D)** Association between the EIF2S2 expression and the T stage of HCC. **(E)** Heatmap of the relationship between EIF2S2 expression and clinicopathological analysis. *p < 0.05, **p < 0.01, ***p < 0.001.

In order to find the correlation between EIF2S2 expression and clinicopathological characteristics of patients with HCC, we analyzed EIF2S2 and clinicopathological characteristics. The results showed that the expression of EIF2S2 was slightly lower in patients over 65 years old (Figure 3A). In pathological grading, the expression of G1 EIF2S2 was significantly different from G3 and G4. Similarly, starting from G2, the expression of EIF2S2 increases with the increase of grading (Figure 3B). There was also a significant difference in EIF2S2 expression between patients with stage I and II, and patients with stage I and III (Figure 3C). In addition, in T stage, the expression of EIF2S2 expression also increased with the increase of T stage (Figure 3D). The heat map of the correlation between EIF2S2 and clinicopathological features also showed the same results (Figure 3E).

### Eukaryotic translation initiation factor 2 subunit beta co-expression analysis

**FIGURE 4:**
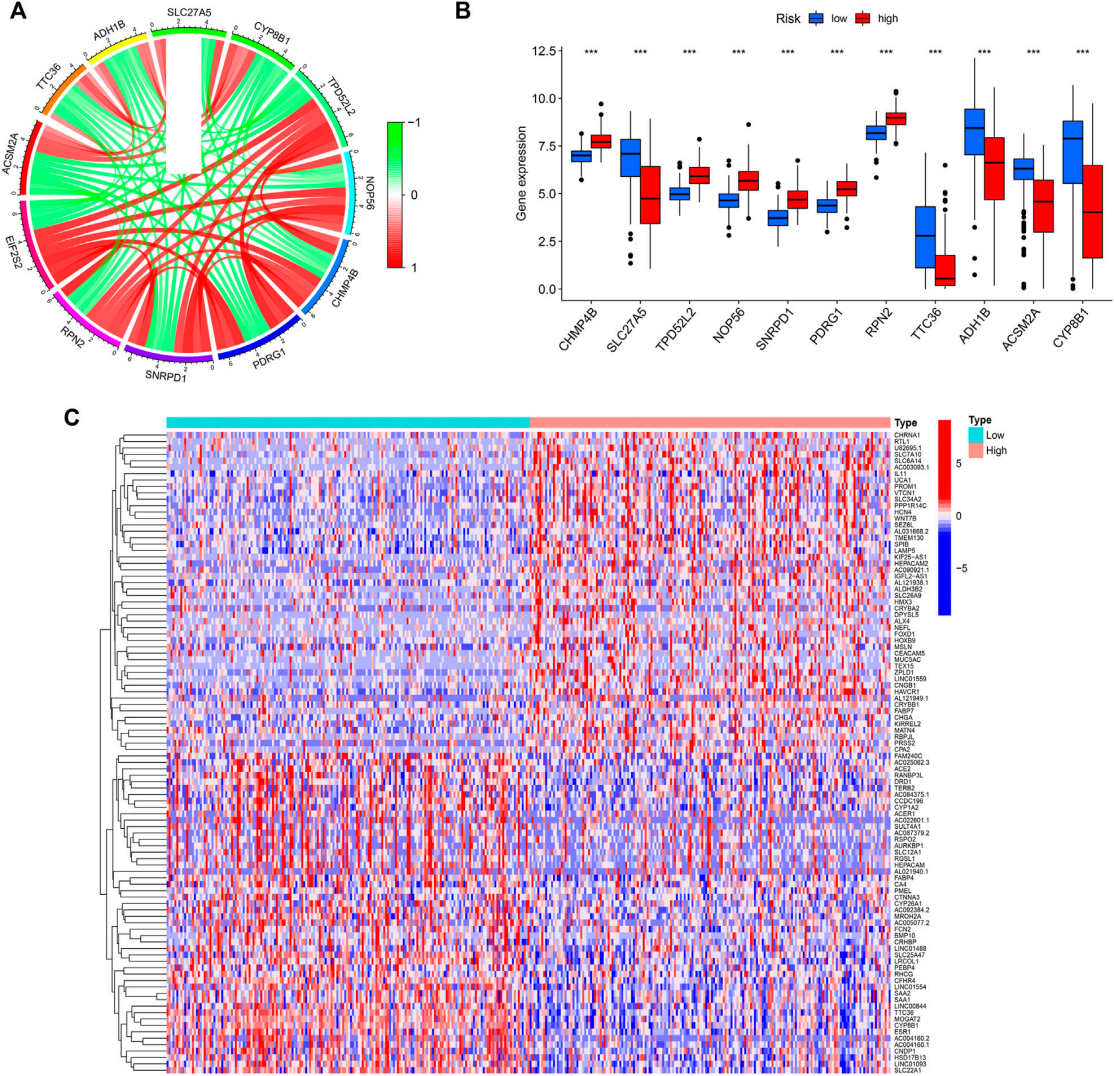
Results of co-expressed analysis. **(A)** Co-expressed genes of EIF2S2. **(B)** Differences in the expression of Co-expressed genes in different expression groups. **(C)** Heatmap of Differential genes.

We screened out the genes co-expressed with EIF2S2 and drew the co-expression circle map. It can be seen from the figure that TPD52L2, NOP56, CHMP4B, PDRG1, SNRPD1, RPN2 and EIF2S2 were positively regulated, while ACSM2A, TTC36, ADH1B, SLC27A5, CYP8B1 and the target gene EIF2S2 were negatively regulated (Figure 4A). Next, we analyzed the relationship between the above genes in the EIF2S2 high expression group and the low expression group. The results showed that the expression of ADH1B, CYP8B1, ACSM2, SLC27A, TTC36 in the low expression group was higher than that in the high expression group, while the expression of other genes was higher in the high expression group (Figure 4B). In addition, we performed differential analysis on genes, and found that CHRNA1, RTL1, U82695.1, etc. were upregulated in the EIF2S2 high expression group, and FAM240C, AC025062.3, ACE2 were elevated in the low expression group of EIF2S2 (Figure 4C).

### Functional enrichment analysis of eukaryotic translation initiation factor 2 subunit beta

**FIGURE 5:**
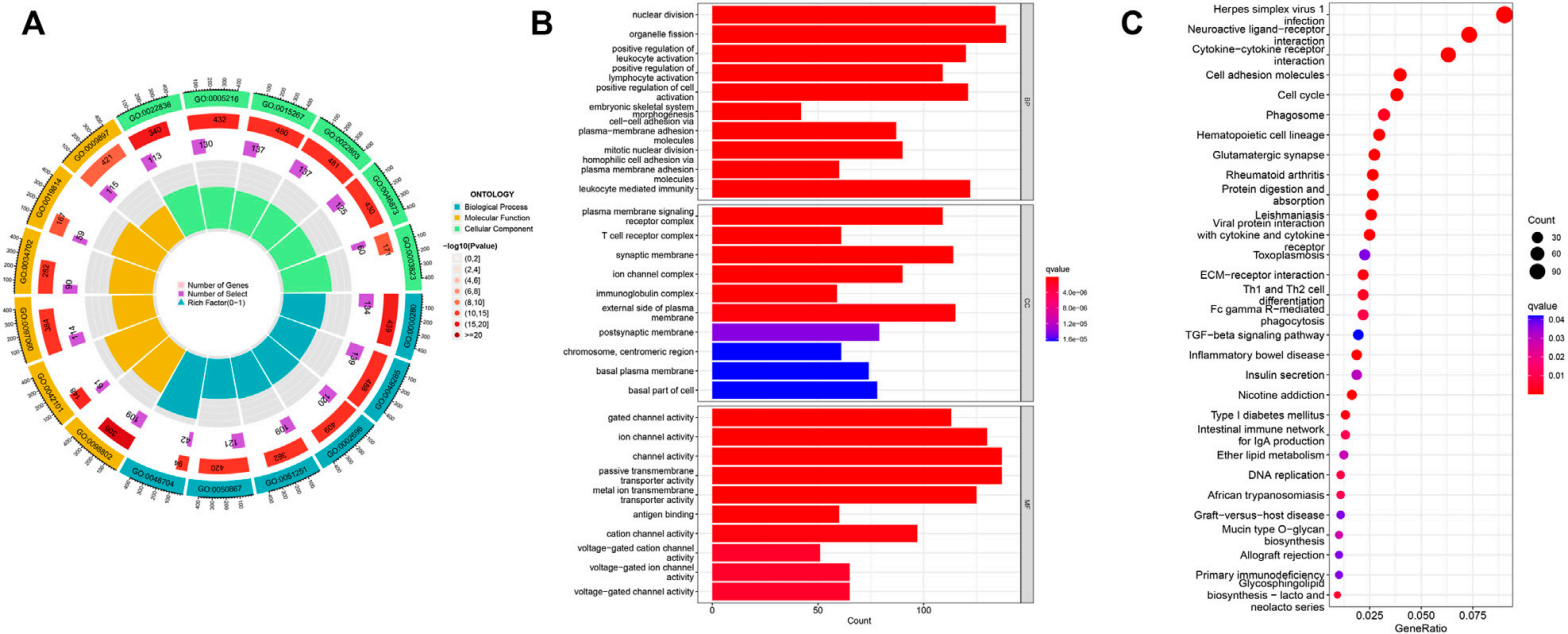
Functional enrichment analysis of EIF2S2 in HCC. **(A,B)** GO terms enrichment analysis. **(C)** KEGG pathway enrichment analysis.

To explore EIF2S2 expression and functional enrichment in the TCGA database, we analyzed the differentially expressed EIF2S2-related genes in HCC cases. We performed GO analysis using the “ClusterProfiler” software package and obtained significantly enriched functions and pathways of EIF2S2 expression. The GO enrichment results show that: EIF2S2 was mainly related to organelle fission, nuclear division and leukocyte mediated immunity in the BP category, associated with external side of plasma membrane, microtubule and synaptic membrane in the CC category, and associated with channel activity, passive transmembrane transporter activity and ion channel activity in the MF category (Figures 5A,B). The KEGG results showed that: EIF2S2 was mainly in neuroactive ligand-receptor interaction, cytokine-cytokine receptor interaction, and cell adhesion molecules, Th1 and Th2 cell differentiation, phagosome and other pathways enriched (Figure 5C). The above results suggest that differential genes regulate many types of biological pathways, participate in a variety of biological processes, and are associated with enriched pathways associated with tumors.

### Correlation between eukaryotic translation initiation factor 2 subunit beta expression and immune cell infiltration

**FIGURE 6:**
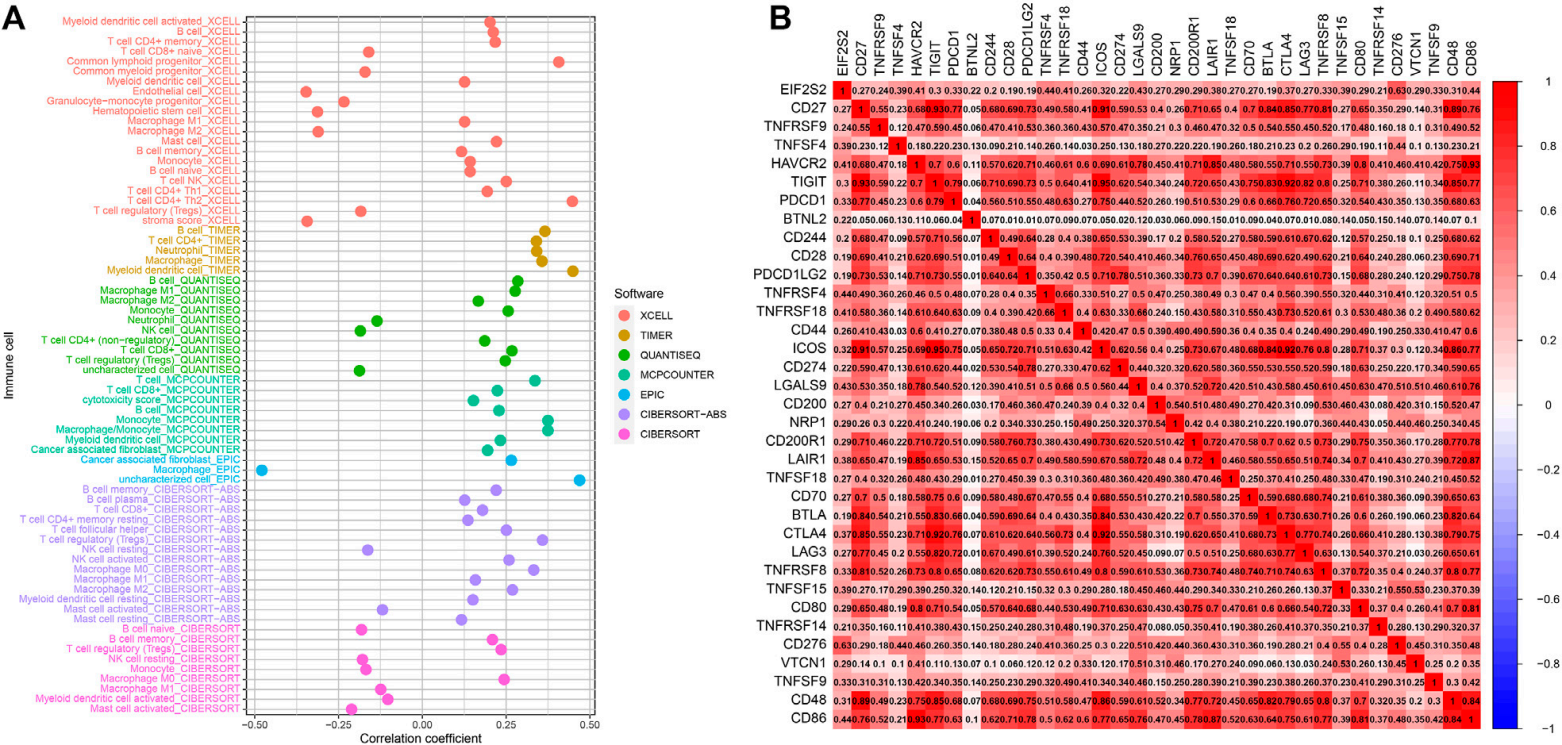
Results of correlation between EIF2S2 expression and immunity. **(A)** Correlation between EIF2S2 expression and immune cell infiltration. **(B)** Correlation of EIF2S2 expression and immune checkpoint genes.

We used multiple algorithms to analyze the relationship of EIF2S2 expression to immune-related cells. The results showed that EIF2S2 expression was positively or negatively correlated with various immune cells. Taking CIBERSORT-ABS as an example, it can be seen that EIF2S2 expression was closely related to memory B cell, plasma B cell, CD8+ T cell, CD4+ resting memory T cell, T follicular helper cells, regulatory T cell, M0 Macrophage, M1 Macrophage, etc. have a positive correlation, and have a negative correlation with resting NK cell and activated mast cell (Figure 6A). At the same time, we also analyzed the relationship between EIF2S2 and immune checkpoint, and the results showed that EIF2S2 expression was positively correlated with PDCD1, TIGIT, CTLA4, LAG-3, BTLA, etc. (Figure 6B).

### Relationship between eukaryotic translation initiation factor 2 subunit beta and drug sensitivity

**FIGURE 7:**
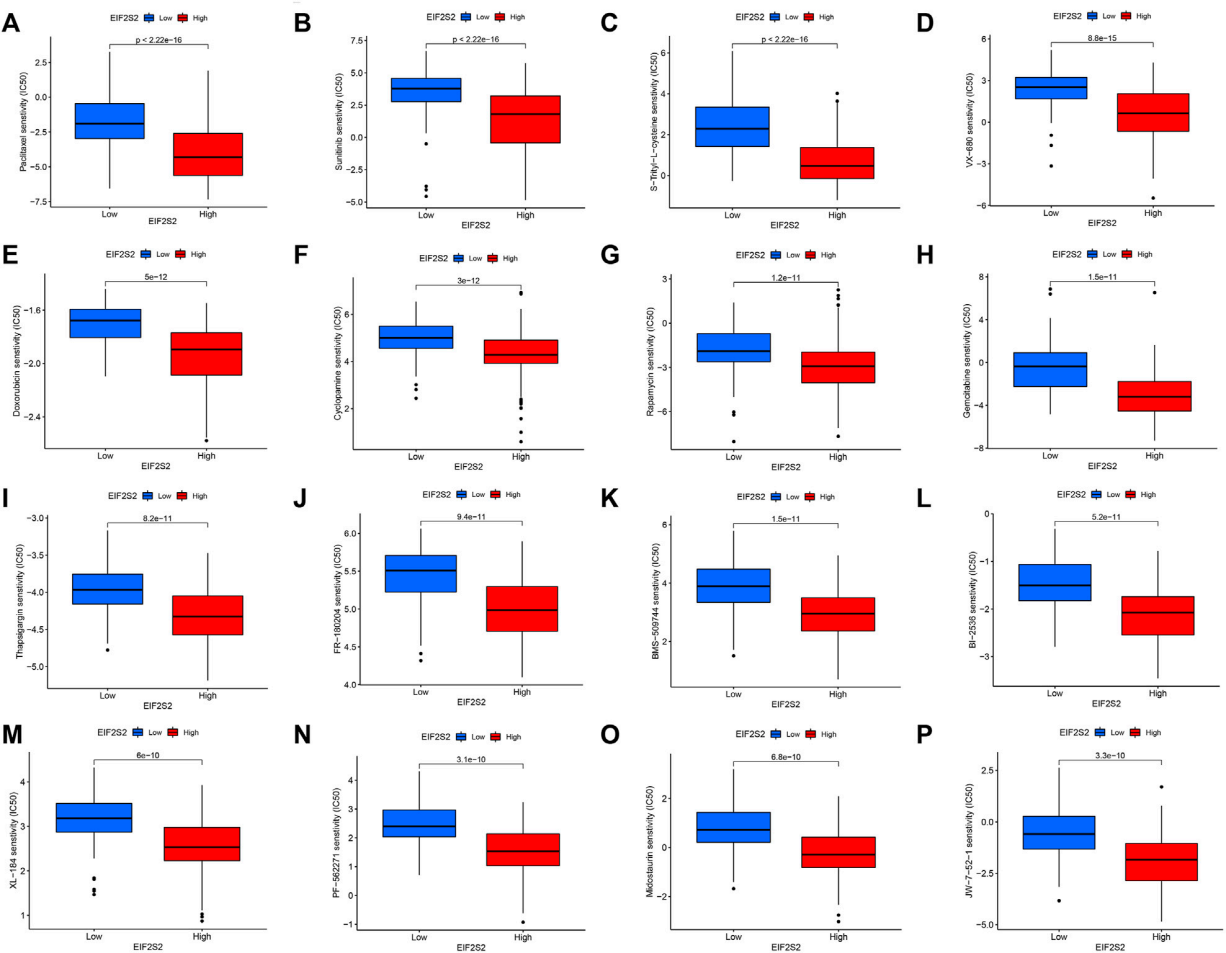
The relationship between EIF2S2 expression levels and drug sensitivity.

Based on the drug sensitivity data from the GDSC database, we predicted the chemosensitivity of each tumor sample by the R software package “pRRophetic” to further explore the correlation between EIF2S2 and common antitumor drug sensitivity. We selected the top 16 drugs with the highest differences. As can be seen from the figure, the EIF2S2 high expression group was more sensitive to Paclitaxel, Sunitinib, S-Trityl-L-cysteine, VX-680, Doxorubicin, Cyclopamine, Rapamycin, and Gemcitabine (Figure 7A−P).

## Discussion

The incidence and mortality from HCC are increasing globally, especially in the United States and Europe, and hepatocellular carcinoma is also one of the fastest rising causes of cancer-related mortality in the United States (Kulik and El-Serag, 2019). Even though surgery may be curative, up to 70% of HCC patients experience recurrence 5 years after surgery (Villanueva, 2019). The main reason for the poor prognosis of HCC is the lack of effective treatments and biomarkers (Liu et al., 2021). Immunotherapies such as immune checkpoint inhibitors have brought new hope for HCC patients (Jiang et al., 2019). However, despite the important role of immunotherapy in the treatment of HCC, identifying clinically useful biomarkers as surrogate indicators of immunotherapy response and prognosis in HCC remains the focus of many *in vitro* and *in vivo* studies (Liu et al., 2022). At present, there is still no simple and effective way to predict the prognosis of HCC patients and provide individualized treatment (Harding et al., 2019).

In our study, we found that EIF2S2 was abnormally expressed in HCC tissues compared to normal tissues, and patients with high EIF2S2 expression had shortened OS and PFS, indicating that EIF2S2 plays an important role in the progression of HCC. The high expression of EIF2S2 predicts poor prognosis of HCC, and both univariate and multivariate COX analysis suggested that high EIF2S2 expression may serve as an independent prognostic factor for HCC. The expression of EIF2S2 is closely related to the clinicopathological features of HCC, such as grade and stage. We found that the high expression of EIF2S2 was correlated with clinicopathological parameters including age, pathological grade, clinical stage, and T stage. In addition, we also found some genes co-expressed with EIF2S2. CHMP4B, TPD52L2 were higher in EIF2S2 high expression group. ADH1B, CYP8B1 in the EIF2S2 low expression group was higher than that in the high expression group. Studies have shown that CHMP4B (Charged multivesicular body protein 4B) is significantly overexpressed in HCC, which is associated with poor prognosis and drug resistance to doxorubicin (Hu et al., 2015). TPD52L2 (tumor protein D52 like 2) regulates the proliferation of hepatocellular carcinoma cells by interacting with ATP binding cassette protein (Zhou et al., 2013). The expression of ADH1B, CYP8B1 was significantly correlated with the good survival of HCC patients (Liu et al., 2020; Zhang et al., 2021). Collectively, EIF2S2 could be serve as a potential prognostic marker for HCC patients.

Through the analysis of GO and KEGG signaling pathways, we found that EIF2S2 was mainly involved in cytokine receptor interactions. It is a key mediator of cell communication in the tumor microenvironment, and cytokines play an important role in the occurrence and development of tumors (Holder et al., 2022). The expression of EIF2S2 was significantly enriched in cytokine receptors, indicating that EIF2S2 was involved in tumor development and immunotherapy. CD4+ helper T (Th) cells are mainly involved in tumor immunology, and can be functionally subdivided into different subgroups, namely Th1, Th2 and Th17 cells, according to the secretion of cytokines and immune function. Th1 cytokines were associated with good prognosis in patients with HCC, while Th2 cytokines are associated with associated with tumor growth or metastasis (Budhu et al., 2006; Mantovani et al., 2008; Zhou et al., 2009). The enrichment results showed that EIF2S2 was enriched with Th1 and Th2 cell pathways, indicating that EIF2S2 was closely related to the prognosis and metastasis of HCC. We also found that EIF2S2 was closely associated with cell adhesion molecules, and down-regulation of EIF2S2 may inhibit the proliferation and migration of HCC through the cell adhesion molecule pathway. Additionally, enriched in phagosomes, cancer cells adapt to a stressful microenvironment by coordinating multiple pathways. Autophagosomes play an important role in the synchronization of internal and external environments during tumorigenesis (Subramanian et al., 2022). Autophagy with dysfunctional autophagosomes has been implicated in a variety of human diseases, including cancer, pathogen infection, diabetes, and neurodegenerative diseases (Mizushima and Levine, 2020).

The liver contains a variety of innate immune cells including NK cells, macrophages, NKT cells, and adaptive immune cells including T cells and B cells, which can affect the status of immune tolerance, tumor progression, and pathogen clearance (Liaskou et al., 2012; Li and Hua, 2017; Ringelhan et al., 2018). Immune infiltration plays an important role in the occurrence and development of HCC (Rohr-Udilova et al., 2018). Tumor immune infiltrating cells are an important part of the tumor microenvironment and have been proven to play an important role in tumor proliferation and metastasis. T cells, B cells, and NK cells are all members of Tumor-Infiltrating Lymphocytes, which are typical components of the host’s antitumor immune response (Ding et al., 2018). CD8+ T lymphocyte dysfunction and exhaustion are characterized by the upregulation of immunosuppressive molecules, such as PD-1, CTLA-4, which inhibit CD8+ T lymphocyte activation. Continued suppression in the tumor microenvironment may result in the inability of infiltrating CD8+ T lymphocytes to kill tumor cells, ultimately leading to tumor immune escape (Schoenberg et al., 2021). In human liver, NK cells can protect hepatocytes from hepatitis virus attack and malignant transformation. The cytotoxicity of NK cells to hepatoma cells can be promoted in several ways. For instance, miR-506 promotes the antitumor effect of NK cells by regulating STAT3 (Su et al., 2019). Our study shows that EIF2S2 is associated with a variety of immune cells, which may promote tumor proliferation and metastasis by promoting immune infiltration of HCC.

In recent years, immune checkpoint inhibitors (ICIs) have made great progress in the treatment of many types of cancer, including HCC(Inarrairaegui et al., 2018). ICIs, including PD-1, PD-L1, and CTLA-4 antibodies, can enhance the activity of effective T cells and suppress immunosuppression in the tumor microenvironment (Kuol et al., 2018). Disappointedly, emerging ICI immunotherapy has been shown to significantly improve clinical outcomes in HCC, but its objective response rate as monotherapy for HCC is only 15%–20% (Ruf et al., 2021). Previous studies have shown that highly expressed PDCD1 has better OS and has a favorable relationship with CD8+ T cells, B cells, macrophages, CD4+ T cells (Li et al., 2022). T cell immunoglobulin and ITIM domain protein (TIGIT) is a type I transmembrane protein, mainly expressed in activated T cells, Treg, memory T cells and NK cells. TIGIT is commonly co-expressed with LAG-3, TIM-3 and PD-1. They are jointly involved in the immune recognition of the body and are closely related to the survival of patients (Anderson et al., 2016). LAG-3 plays an important role in negatively regulating T cell activation and proliferation. It is expected to become a major target after PD-1 in the development of cancer therapy (Maruhashi et al., 2020). B and T lymphocyte attenuator (BTLA) is a lymphocyte inhibitory receptor similar to CTLA-4 and PD-1. BTLA is a marker that recognizes exhausted PD-1-expressing CD4 T cells and can functionally inhibit CD4 T lymphocyte function through interaction with herpesvirus entry mediator cells (Zhao et al., 2016). Regarding immune checkpoints associated with tumor cell immune evasion, such as CTLA-4, PD-1, PD-L1, TIGIT, these inhibitory receptors/ligands suppress antitumor immune responses by altering their expression levels (Xing et al., 2021). In this study, the expression of EIF2S2 was significantly correlated with the expression of the above-mentioned immune checkpoints, which is of great significance. These findings may provide new ideas and research prospects for EIF2S2 in the treatment of HCC in clinical settings.

Paclitaxel is a broadly active and potent cytotoxic drug. Hepatic arterial infusion of paclitaxel has good efficacy in patients with advanced colorectal cancer, thyroid cancer and hepatocellular carcinoma with liver metastases (Tsimberidou et al., 2011). Sunitinib is a multi-targeted receptor tyrosine kinase inhibitor with antitumor activity (Nassif et al., 2017). Studies have shown that transarterial chemoembolization plus sunitinib is safe and feasible for patients with liver cancer who are not suitable for surgical resection (Turpin et al., 2021). Sunitinib combined with Minimally invasive radiofrequency ablation can significantly inhibit the growth of liver cancer (Qi et al., 2020). Doxorubicin is an antitumor antibiotic that inhibits the synthesis of RNA and DNA, and has effects on a variety of tumors. The chitosan-coated doxorubicin nanoparticle drug delivery system can inhibit the growth of hepatoma cells through the p53/PRC1 pathway (Ye et al., 2018). S-triphenyl-L-cysteine is an Eg5 inhibitor that blocks cell mitosis and exhibits tumor growth inhibitory activity (Kozielski et al., 2008). Our results show that the IC50 of the EIF2S2 high expression group is lower than that of the low expression group, and the sensitivity to these drugs is higher.

This study is the first to our knowledge to have explored the relationship between EIF2S2 and outcomes and immune infiltration in HCC patient. However, our study also has certain limitations and needs to be confirmed *in vivo* and *in vitro* experiments. In the future, more immunosuppressants need to be developed, new treatments explored, and new prognostic biomarkers discovered for better therapeutic effects.

## Conclusion

EIF2S2 is a novel biomarker that can predict the prognosis of HCC, and its high expression is correlated with the OS, PFS and clinicopathological characteristics of HCC patients. In addition, EIF2S2 is also associated with immune cell infiltration and immune checkpoint, which can become a potential therapeutic approach, bringing new hope for the clinical treatment of HCC (AuthorAnonymous et al., 2022).

## Data Availability

The datasets presented in this study can be found in online repositories. The names of the repository/repositories and accession number(s) can be found in the article/Supplementary Material.
